# Prevalence and prognostic significance of cardiac autonomic neuropathy in community-based people with type 2 diabetes: the Fremantle Diabetes Study Phase II

**DOI:** 10.1186/s12933-024-02185-3

**Published:** 2024-03-18

**Authors:** Timothy M. E. Davis, Eva Tan, Wendy A. Davis

**Affiliations:** 1 Medical School, Fremantle Hospital, University of Western Australia, PO Box 480, Fremantle, WA 6959 Australia; 2 Department of Endocrinology and Diabetes, Fiona Stanley and Fremantle Hospitals, Murdoch, WA Australia; 3 Australian Centre for Accelerating Diabetes Innovations (ACADI), The University of Melbourne, Melbourne, VIC Australia

**Keywords:** Type 2 diabetes, Cardiac autonomic neuropathy, Ischemic heart disease, Heart failure, Mortality

## Abstract

**Background:**

There is a paucity of contemporary data on the prevalence and prognostic significance of cardiac autonomic neuropathy (CAN) from community-based cohorts with type 2 diabetes assessed using gold standard methods. The aim of this study was to assess these aspects of CAN in the longitudinal observational Fremantle Diabetes Study Phase II (FDS2).

**Methods:**

FDS2 participants were screened at baseline using standardised cardiovascular reflex tests (CARTs) of heart rate variation during deep breathing, Valsalva manoeuvre and standing. CAN (no/possible/definite) was assessed from the number of abnormal CARTs. Multinomial regression identified independent associates of CAN status. Cox proportional hazards modelling determined independent baseline predictors of incident heart failure (HF) and ischaemic heart disease (IHD), and all-cause mortality.

**Results:**

Of 1254 participants assessed for CAN, 86 (6.9%) were outside CART age reference ranges and valid CART data were unavailable for 338 (27.0%). Of the remaining 830 (mean age 62.3 years, 55.3% males, median diabetes duration 7.3 years), 51.0%, 33.7% and 15.3% had no, possible or definite CAN, respectively. Independent associates of definite CAN (longer diabetes duration, higher body mass index and resting pulse rate, antidepressant and antihypertensive therapies, albuminuria, distal sensory polyneuropathy, prior HF) were consistent with those reported previously. In Kaplan–Meier analysis, definite CAN was associated with a lower likelihood of incident IHD and HF versus no/possible CAN (*P* < 0.001) and there was a graded increase in all-cause mortality risk from no CAN to possible and definite CAN (*P* < 0.001). When CAN category was added to the most parsimonious models, it was not a significant independent predictor of IHD (*P* ≥ 0.851) or HF (*P* ≥ 0.342). Possible CAN (hazard ratio (95% CI) 1.47 (1.01, 2.14), *P* = 0.046) and definite CAN (2.42 (1.60, 3.67), *P* < 0.001) increased the risk of all-cause mortality versus no CAN.

**Conclusions:**

Routine screening for CAN in type 2 diabetes has limited clinical but some prognostic value.

**Supplementary Information:**

The online version contains supplementary material available at 10.1186/s12933-024-02185-3.

## Background

Cardiovascular autonomic neuropathy (CAN) is characterised by orthostatic hypotension, resting tachycardia, impaired exercise tolerance and abnormal blood pressure regulation [1], but it may also remain asymptomatic and thus elude timely diagnosis [2]. It is a common chronic complication of type 2 diabetes with a prevalence estimated at between 9 and 78% from studies conducted in primary but mainly secondary care [3, 4]. Among a number of available CAN diagnostic tests, the gold standard comprises several standardised cardiovascular reflex tests (CARTs) including the electrocardiographic R-R interval response to deep breathing, the Valsalva manoeuvre and postural changes in blood pressure [5]. CARTs are, however, not widely available, time-consuming, and difficult to perform in people with mobility challenges and in whom forceful breathing is difficult or even contra-indicated [6].

These considerations may underlie the wide range of CAN prevalence estimates in type 2 diabetes, but may also have implications for assessment of the relationship between CAN and both cardiovascular disease (CVD) and death. A recent meta-analysis of unadjusted data suggested that CAN increases the risk of CVD events and all-cause mortality in type 2 diabetes more than threefold [7], but there was substantial heterogeneity between studies. Indeed, the risk of all-cause death in the high CVD risk Action to Control Cardiovascular Risk in Diabetes (ACCORD) trial sample was lower, if still significantly increased, in fully adjusted statistical models [8]. Furthermore, CVD risk factors are closely associated with the development of CAN [3]. A number of studies included in the meta-analysis were conducted before the publication of the results of intervention trials supporting more intensive management of hypertension and dyslipidaemia in type 2 diabetes which have resulted in improved CVD risk factor management [9, 10] and a reduction in CVD events [11–14] over recent decades.

Although testing for autonomic dysfunction has been recommended as part of routine early screening in diabetes [2, 7], its role as an independent predictor of CVD events and mortality needs to be established in contemporary cohorts of people with type 2 diabetes. The aim of this study was, therefore, to assess the prevalence and prognostic significance of CAN in well characterised, representative, community-based participants from the Fremantle Diabetes Study Phase II (FDS2).

## Methods

### Study site, participants and approvals

The FDS2 is an observational study conducted in a postcode-defined urban community of 157,000 people in the state of Western Australia (WA) [15]. Socio-economic data relating to income, employment, housing, transportation and other variables in the study area show an average Index of Relative Socio-economic Advantage and Disadvantage of 1033 with a range by postcode of 977 to 1113, figures similar to the Australian national mean ± SD of 1000 ± 100 [16]. Descriptions of FDS2 recruitment, sample characteristics and details of non-recruited people with diabetes have been published [15]. Individuals resident in the catchment area with a clinician-verified diagnosis of diabetes (excluding gestational diabetes) were identified through available hospital and community sources. Of 4639 with known diabetes found between 2008 and 2011, 1668 (36.0%) were recruited. Sixty-four FDS Phase I participants recruited between 1993 and 1996 who had moved out of the catchment area were also enrolled (total cohort 1732, of whom 1551 had clinically diagnosed type 2 diabetes). For the purposes of the present study, there were 1254 participants (89.9% of the FDS2 type 2 diabetes cohort) who were eligible for CAN testing after it first became available in May 2009 as part of baseline assessment.

### Clinical and laboratory assessments

All FDS2 participants were invited to face-to-face assessments at entry and then biennially [15]. Each assessment included a standardised comprehensive questionnaire and physical examination, and fasting biochemical tests performed in a single nationally accredited laboratory. Participants were requested to bring all medications and/or prescriptions to each visit. Racial/ethnic background was categorised based on self-selection, country/countries of birth and parents’/grandparents’ birth, and language(s) spoken at home as Anglo-Celt, Southern European, Other European, Asian, Aboriginal or mixed/other. Body mass index (BMI) was determined together with a body shape index (ABSI) which represents a more reliable estimate of visceral adiposity [17]. Orthostatic hypotension was defined as a fall in systolic blood pressure of ≥ 20 mmHg or in diastolic blood pressure of ≥ 10 mmHg within three minutes of standing [18].

The CARTs were performed on each eligible participant in the morning after an overnight fast, and comprised measurement and analysis of heart rate variation during deep breathing, the Valsalva manoeuvre, and on standing by electrocardiography using the ANS 2000 system (Hokanson Inc, Bellevue, Washington, US) [19–21]. The deep breathing test was performed with the participant supine and breathing at a paced rate of five breaths/minute for six minutes, as recommended by the manufacturer. The ratio of the mean of the shortest R-R interval during inspiration to the mean of the longest R-R wave during expiration (E:I ratio) was calculated, and the MCR determined by vector analysis of the R–R intervals [22]. During the Valsalva manoeuvre, participants performed continuous forced exhalation to a pressure of 40 mmHg for 15 s. The ratio of the longest R–R interval after the manoeuvre to the shortest R–R interval during the manoeuvre was calculated (the Valsalva ratio) [21]. Evaluation of changes in heart rate was performed during the initial phase of adaptation to orthostasis (first 45 s), and the 30:15 stand ratio calculated from the maximal (around 30th heart beat) to minimal (near 15th heart beat) R-R interval [22].

Abnormalities in the three CART components (one or other of E:I ratio and MCR in the case of the deep breathing CART) were identified from age-corrected normal ranges [22] and given a score of 1. Since the age of the healthy individuals used to derive the normal ranges spanned 15–67 years [22], linear (for E:I ratio, MCR and Valsalva ratio; r^2^ ≥ 0.983) and quadratic (for 30:15 stand ratio; r^2^ = 0.996) equations were derived from the table of age versus the 2.3 centiles of each CART [22] and extrapolated to age 80 years to better capture reflect the age range of the FDS2 type 2 diabetes cohort and to parallel other studies with age-specific reference ranges which did not include very elderly participants [23, 24]. Diagnosis of CAN and its stage was determined from modified Toronto Consensus Panel criteria [25] as no CAN (total score = 0), possible CAN (total score = 1) or definite CAN (total score ≥ 2).

Chronic complications of diabetes were identified using standard definitions [15]. Albuminuria was assessed by early morning spot urinary albumin:creatinine ratio (uACR) measurement and renal impairment from the estimated glomerular filtration rate (eGFR) [26]. Distal symmetrical polyneuropathy (DSPN) was defined using the vibration perception threshold [27]. Retinopathy was defined as one microaneurysm in either eye or worse and/or previous laser treatment on fundus photography and/or ophthalmologist assessment. Peripheral arterial disease (PAD) was defined as an ankle brachial index ≤ 0.90 or a diabetes-related lower extremity amputation.

### Ascertainment of cardiovascular outcomes and deaths

The Hospital Morbidity Data Collection (HMDC) contains validated information regarding all public/private hospitalisations in WA since 1970 and the Death Register contains information on all deaths in WA [28]. The FDS2 database has been linked to these databases through the WA Data Linkage System (WADLS), as approved by the WA Department of Health Human Research Ethics Committee. The HMDC was used to supplement data obtained through FDS assessments relating to prevalent/prior complications/conditions during the five years prior to study entry. A prior history of ischaemic heart disease (IHD), cerebrovascular disease or heart failure (HF) were defined as hospitalisations or death with/for/of IHD, cerebrovascular disease or HF, respectively, before the first CAN assessment. Incident IHD was defined as hospitalisations or death with/for/of IHD or cardiac/sudden death, and incident HF as hospitalisations or death with/for/of HF, both endpoints being ascertained from the first CAN assessment to end-December 2021. Causes of death on the death certificate or coroner’s report were reviewed independently by two study physicians and classified under the system used in the UK Prospective Diabetes Study [11]. In the case of discrepant coding, case notes were consulted and a consensus obtained. Death from IHD was defined as death from non-HF cardiac or sudden death, and death from HF was defined as cardiac death in which HF dominated. All endpoints were ascertained from the first CAN assessment to end-December 2021.

### Statistical analysis

The computer packages IBM SPSS Statistics 29 (IBM Corporation, Armonk, NY, USA) and StataSE 15 (College Station, TX: StataCorp LP) were used for statistical analysis. Data are reported as percentage, mean ± SD, geometric mean (SD range), or, when variables are not normally distributed, median [interquartile range]. Two-way comparisons were performed using Fisher’s exact test for independent samples, the normally distributed variables compared using Student’s *t*-test, and the non-normally distributed variables using Mann–Whitney U-test. Comparisons between multiple groups for categoric variables were by Fisher-Freeman-Halton exact or Chi-squared tests, for normally/log-normally distributed continuous variables by one-way ANOVA, and for variables not conforming to normal/log-normal distribution by Kruskal–Wallis test. Where the overall trend for these multiple comparisons was statistically significant, *post-hoc* Bonferroni-corrected pairwise comparisons were performed.

Multinomial regression was used to identify independent associates of CAN status with the no CAN group as reference. Clinically relevant and biologically plausible variables with bivariable *P* < 0.20 were considered for model entry. Variables were removed sequentially if *P* ≥ 0.050 for every CAN group (relative to the reference category), the least significant being removed first, until all variables in the model were significant in at least one group.

Cox proportional hazards modelling (backward conditional variable selection with *P* < 0.050 for entry and ≥ 0.050 for removal) was used to determine independent baseline predictors of incident HF and IHD, and all-cause mortality. All clinically plausible variables with bivariable *P* ≤ 0.20 were considered for entry into these models in a backward stepwise manner and included demographic and diabetes-related factors, the presence of other complications and cardiovascular risk factors. Aboriginal status was also considered for entry since Aboriginal participants were significantly younger than other ethnic groups. After the most parsimonious model in each instance was defined, CAN status was entered. The proportional hazards assumption was assessed and, if violated, adjusted for by adding significant time-varying covariates. A two-tailed significance level of *P* < 0.05 was used throughout.

## Results

### Baseline participant characteristics

Of the 1254 FDS2 participants who underwent CAN testing at baseline, 86 (6.9%) were excluded because they were aged < 20 years or > 80 years and so their CART data could not be assessed against extrapolated age-specific normal ranges [22]. Of the remaining 1168 participants, a further 338 (27.0%) were excluded because they could not perform all the tests according to protocol, they had poor quality electrocardiographic recordings that were unsuitable for analysis, or they had a significant cardiac arrhythmia that confounded interpretation of the results. Compared to the 830 with complete CART data required for CAN categorisation, the 424 who were excluded were significantly older (age 62.3 ± 10.5 versus 71.0 ± 10.6 years, *P* < 0.001), less likely to be males (56.0% versus 45.8%, *P* < 0.001), had longer diabetes duration (7.1 versus 11.2 years, *P* < 0.001), and were significantly more likely to have chronic complications (see Additional file 1: Table S1).

The baseline characteristics of included participants are summarised by CAN status in Table 1. Approximately 15% had definite CAN, one third had possible CAN and around one half had no CAN. Compared with the other two groups, those with definite CAN were more likely to have an Aboriginal background, to be diagnosed with diabetes at a younger age and to have longer diabetes duration, to be obese, and to have a higher HbA_1c_ despite a greater likelihood of insulin therapy. They were also more likely to be treated with antidepressants, to have a higher resting pulse rate in the presence of greater beta blocker and calcium channel blocker use, to have hypertriglyceridemia and microalbuminuria, to have greater degrees of renal impairment, to have DSPN, and to have a prior history of IHD and HF. Those with possible CAN had diabetes duration, and prevalences of IHD and HF, that were intermediate between those in the no CAN and definite CAN groups.

**Table 1:** Baseline characteristics of FDS2 participants categorised by CAN status

	No CAN	Possible CAN	Definite CAN	*P*-value
N (%)	423 (51.0)	280 (33.7)	127 (15.3)	
Orthostatic hypotension (%)	12.3	11.5	11.1	0.945
Age (years)	62.2 ± 10.5	62.1 ± 10.2	63.2 ± 11.4	0.555
Male (%)	57.9	54.6	52.8	0.497
Education beyond primary level (%)	92.3	94.2	90.3	0.366
Not fluent in English (%)	6.6	7.1	6.3	0.946
Ethnic background (%)				0.053
Anglo-Celt	55.1	51.4	48.8	
Southern European	10.6	11.1	10.2	
Other European	5.9	8.9	10.2	
Asian	4.0	5.7	3.1	
Aboriginal	5.2	10.0	11.8	
Mixed/other	19.1	12.9	15.7	
Smoking status (%)				0.262
Never	53.5	50.9	50.4	
Ex-	37.7	36.5	34.1	
Current	8.8	12.6	15.4	
Alcohol use (standard drinks^a^/day)	0.3 [0–1.5]	0.3 [0–1.5]	0.1 [0–1.0]	0.455
Antidepressant use (%)	10.6	16.4	24.4^***^	< 0.001
Tricyclic antidepressants	2.6	1.8	8.7^*,†^	0.003
Selective serotonin reuptake inhibitors	6.1	11.1	7.1	0.061
Age at diabetes diagnosis (years)	53.8 ± 11.0	53.1 ± 10.7	49.4 ± 13.9^***,††^	< 0.001
Diabetes duration (years)	6.0 [2.6–11.9]	8.0 [3.0–15.1]	10.0 [4.0–17.2]^***,†††^	< 0.001
Diabetes treatment (%)			^***,†††^	< 0.001
Diet/exercise alone	26.5	22.7	12.7	
Oral glucose lowering agents	55.7	55.6	46.0	
Insulin alone	3.3	2.5	8.7	
Insulin + oral agents	14.5	19.1	32.5	
HbA_1c_ (%)	6.8 [6.2–7.8]	6.9 [6.2–7.8]	7.3 [6.6–8.4]^***,††^	< 0.001
HbA_1c_ (mmol/mol)	50 [44–58]	51 [44–61]	57 [49–74]	< 0.001
Fasting serum glucose (mmol/L)	7.2 [6.3–8.6]	7.4 [6.4–9.3]	8.2 [6.7–10.3]^***,†^	< 0.001
ABSI (m^11/6^ kg^−2/3^)	0.081 ± 0.005	0.081 ± 0.005	0.082 ± 0.005	0.051
BMI (kg/m^2^)	31.2 ± 5.9	32.4 ± 6.2^*^	33.5 ± 7.1^**^	< 0.001
Heart rate (beats/min)	69 ± 11	70 ± 11	74 ± 13^***,††^	< 0.001
Supine systolic blood pressure (mmHg)	141 ± 19	143 ± 21	143 ± 22	0.478
Supine diastolic blood pressure (mmHg)	81 ± 11	81 ± 11	78 ± 14	0.071
Antihypertensive medication (%)	66.0	72.1	78.7^*^	0.013
Angiotensin converting enzyme inhibitors	32.9	33.6	38.6	0.479
Angiotensin receptor blockers	30.5	36.4	38.6	0.119
Beta-blockers	10.9	21.4^***^	29.9^***^	< 0.001
Calcium channel blockers	16.8	26.1^**^	36.2^***^	< 0.001
Diuretics	20.1	29.3^*^	37.8^***^	< 0.001
Total serum cholesterol (mmol/L)	4.3 ± 0.9	4.4 ± 1.1	4.5 ± 1.6	0.215
Serum HDL-cholesterol (mmol/L)	1.21 ± 0.32	1.16 ± 0.28	1.17 ± 0.32	0.064
Serum triglycerides (mmol/L)	1.5 (0.9–2.4)	1.6 (0.9–2.7)	1.7 (1.1–2.8)^**^	0.004
Lipid-lowering medication (%)	67.8	67.5	69.3	0.947
uACR (mg/mmol)	2.2 (0.7–6.7)	2.9 (0.8–10.4)^*^	5.2 (0.9–30.5)^***,†††^	< 0.001
eGFR (mL/min/1.73m^2^)	85 ± 17	84 ± 20	77 ± 23^***,††^	< 0.001
eGFR categories (%)		^*^	^***,†^	< 0.001
≥ 90 mL/min/1.73m^2^	43.0	47.9	30.7	
60–89 mL/min/1.73m^2^	49.9	39.6	44.9	
45–59 mL/min/1.73m^2^	5.2	6.4	13.4	
30–44 mL/min/1.73m^2^	1.4	4.3	6.3	
< 30 mL/min/1.73m^2^	0.5	1.8	4.7	
Distal symmetrical polyneuropathy (%)	29.4	32.1	44.4^**^	0.007
Peripheral arterial disease (%)	18.2	20.1	27.6	0.076
Prior IHD hospitalisation (%)	15.1	23.2^*^	32.3^***^	< 0.001
Prior cerebrovascular disease hospitalisation (%)	4.5	4.6	7.9	0.309
Prior HF hospitalisation (%)	1.7	5.7^*^	8.7^**^	< 0.001

^a^1 standard drink = 10 U ethanol; *P < 0.05, **P < 0.01, ***P < 0.001 vs no CAN, ^†^*P* < 0.05, ^††^*P* < 0.01, ^†††^*P* < 0.001 vs possible CAN in Bonferroni-corrected multiple comparisons

The independent associates of CAN group identified by multinomial modelling are shown in Table 2. Compared to the group without CAN, those with possible or definite CAN were more likely to be treated with beta blockers, calcium channel blockers and antidepressants, and to have a history of HF. In addition, those with possible CAN were more likely to be Aboriginal, while those with definite CAN were had a higher BMI and resting pulse rate, longer diabetes duration, a greater uACR, and a higher likelihood of DSPN.

**Table 2:** Independent associates of CAN category in FDS2 participants with type 2 diabetes

	Possible CAN	Definite CAN
	OR (95% CI)	OR (95% CI)
Aboriginal descent	1.91 (1.01, 3.62)	1.65 (0.72, 3.78)
Diabetes duration (increase of 1 year)	1.01 (0.98, 1.03)	1.05 (1.02, 1.08)
Body mass index (increase of 1 kg/m^2^)	1.02 (0.99, 1.05)	1.04 (1.005, 1.08)
Pulse rate (increase of 1 beat/min)	1.01 (0.996, 1.03)	1.04 (1.02, 1.06)
On beta-blockers	1.95 (1.24, 3.09)	2.86 (1.60, 4.93)
On calcium channel blockers	1.70 (1.15, 2.50)	2.44 (1.47, 4.04)
On antidepressant medication	1.67 (1.06, 2.64)	2.81 (1.60, 4.93)
Ln [urinary albumin:creatinine ratio (mg/mmol)]^a^	1.09 (0.95, 1.24)	1.29 (1.10, 1.53)
Distal symmetrical polyneuropathy	1.12 (0.79, 1.58)	1.65 (1.04, 2.61)
Prior hospitalisation for heart failure	2.96 (1.11, 7.92)	3.46 (1.15, 10.4)

Multinomial regression was used with no prevalent CAN as the reference. Data are odds ratios (OR) and 95% confidence intervals (CIs). Values are provided for both CAN categories where variables were significant for at least category

^a^A 2.72-fold increase in urinary albumin:creatinine ratio corresponds to an increased risk of 1 in ln(urinary albumin:creatinine)

### Incident ischaemic heart disease

**Fig. 1:**
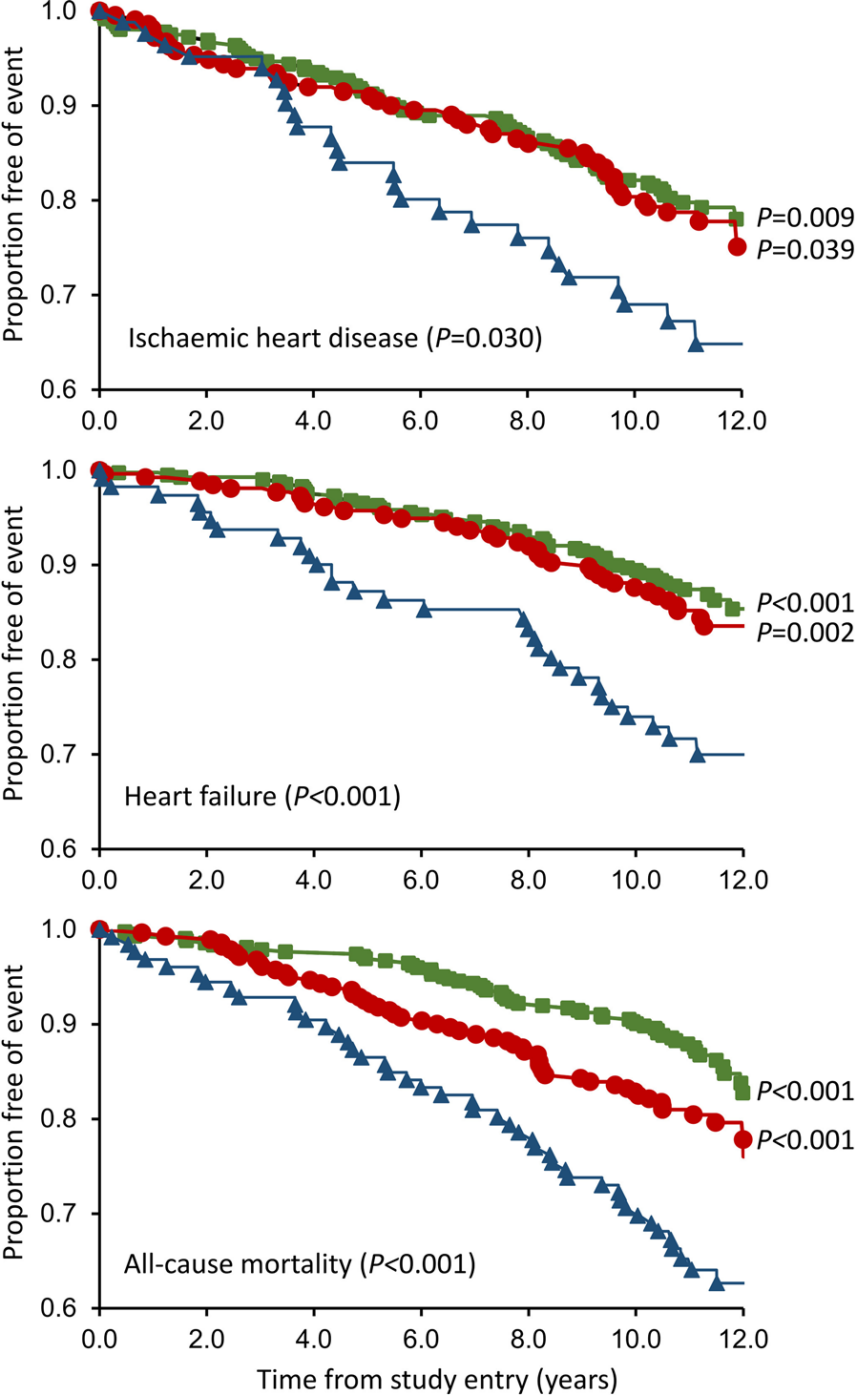
Kaplan–Meier plots of incident IHD, HF and all-cause mortality for FDS2 participants with no CAN (green square), possible CAN (red circle) and definite CAN (blue up-pointing triangle). *P*-values are from log-rank tests

The characteristics of the 142 FDS2 participants (21.6%) who had an IHD event during follow-up and those who did not are summarised in Table 3. The Kaplan–Meier curves of IHD events during a mean ± SD follow-up period of 9.7 ± 3.2 (range 0.0–12.6) years (equivalent to 6354 person-years) are shown in Fig. 1 (upper panel). There was a statistically significant difference between the three groups (log rank test *P* = 0.030) with definite CAN significantly different from both the no CAN group (*P* = 0.009) and possible CAN group (*P* = 0.039) in unadjusted pairwise comparisons. In Cox proportional hazards modelling, longer diabetes duration, a higher heart rate and uACR, and DSPN were independent predictors of incident IHD events, but CAN category did not add significantly to the model (see Table 4).

**Table 3:** Baseline characteristics of FDS2 participants with type 2 diabetes by incident IHD status, excluding those with pre-recruitment hospitalisation for/with IHD

	No hospitalisation for/with IHD or non-HF cardiac/sudden death	Incident hospitalisation for IHD or non-HF cardiac/sudden death^b^	*P*-value
N (%)	516 (78.4)	142 (21.6)	
CAN group (%)			0.099
None	55.6	50.0	
Possible	32.9	31.7	
Definite	11.4	18.3	
MCR (n = 658)	21.4 [12.4–34.8]	22.1 [11.9–31.0]	0.578
E:I ratio (n = 658)	1.13 [1.09–1.20]	1.13 [1.08–1.18]	0.198
Valsalva ratio (n = 642)	1.51 [1.33–1.73]	1.44 [1.28–1.58]	0.009
30:15 Stand ratio (n = 657)	1.16 [1.09–1.25]	1.12 [1.07–1.21]	< 0.001
Orthostatic hypotension (%)	14.8	8.5	0.069
Age (years)	60.9 ± 10.5	62.0 ± 10.5	0.257
Male (%)	53.1	54.9	0.705
Education beyond primary level (%)	93.7	92.9	0.702
Not fluent in English (%)	6.4	6.3	> 0.999
Ethnic background (%)			0.046
Anglo-Celt	54.1	50.0	
Southern European	11.0	10.6	
Other European	7.2	7.7	
Asian	4.8	2.8	
Aboriginal	5.6	14.1	
Mixed/other	17.2	14.8	
Smoking status (%)			0.225
Never	54.5	46.4	
Ex-	34.9	40.7	
Current	10.6	12.9	
Alcohol consumption (standard drinks^a^/day)	0.3 [0–1.5]	0.1 [0–0.8]	0.034
Antidepressant use (%)	14.1	15.5	0.687
Tricyclic antidepressants	2.5	4.2	0.267
Selective serotonin reuptake inhibitors	8.9	6.3	0.394
Age at diabetes diagnosis (years)	52.8 ± 11.2	50.9 ± 12.3	0.076
Diabetes duration (years)	5.7 [2.6–13.0]	10.0 [4.2–16.1]	< 0.001
Diabetes treatment (%)			0.019
Diet/exercise	25.5	17.0	
Oral agents ± non-insulin injectables	56.1	53.9	
Insulin alone	2.7	5.0	
Insulin ± oral agents ± non-insulin injectables	15.6	24.1	
HbA_1c_ (%)	6.8 [6.2–7.6]	7.1 [6.3–8.3]	0.005
HbA_1c_ (mmol/mol)	51 [44–60]	54 [45–67]	0.005
Fasting serum glucose (mmol/L)	7.3 [6.4–8.9]	7.6 [6.5–10.6]	0.035
ABSI (m^11/6^ kg^−2/3^)	0.080 ± 0.005	0.082 ± 0.005	0.004
BMI (kg/m^2^)	32.2 ± 6.2	31.8 ± 7.0	0.502
Pulse rate (beats/min)	70 ± 11	73 ± 12	0.004
Supine systolic blood pressure (mmHg)	141 ± 20	146 ± 20	0.003
Supine diastolic blood pressure (mmHg)	81 ± 12	81 ± 13	0.851
Antihypertensive medication (%)	64.3	65.5	0.843
Angiotensin converting enzyme inhibitors	30.2	33.1	0.539
Angiotensin receptor blockers	31.2	35.2	0.363
Beta-blockers	8.9	9.9	0.743
Calcium channel blockers	20.2	21.1	0.814
Diuretics	24.0	28.9	0.274
Total serum cholesterol (mmol/L)	4.3 ± 1.0	4.5 ± 1.2	0.040
Serum HDL-cholesterol (mmol/L)	1.21 ± 0.33	1.19 ± 0.29	0.534
Serum triglycerides (mmol/L)	1.5 (0.9–2.4)	1.6 (1.0–2.7)	0.028
Lipid-lowering medication (%)	64.1	61.3	0.555
uACR (mg/mmol)	2.3 (0.7–7.5)	4.1 (0.8–21.6)	< 0.001
eGFR (mL/min/1.73m^2^)	86 ± 18	82 ± 21	0.048
eGFR categories (%)			0.254
≥ 90 mL/min/1.73m^2^	47.2	41.1	
60–89 mL/min/1.73m^2^	43.7	44.7	
45–59 mL/min/1.73m^2^	6.0	7.8	
30–44 mL/min/1.73m^2^	1.7	4.3	
< 30 mL/min/1.73m^2^	1.4	2.1	
Distal symmetrical polyneuropathy (%)	28.7	38.3	0.031
Peripheral arterial disease (%)	17.9	23.2	0.184
Prior hospitalisation for cerebrovascular disease (%)	2.9	6.3	0.073
Prior hospitalisation for HF (%)	1.4	2.8	0.264

^a^1 standard drink = 10 U ethanol; ^b^three participants with unknown cause of death excluded from this analysis

**Table 4:** Most parsimonious Cox models of independent predictors of IHD, HF and all-cause death in the FDS2 cohort with CAN category defined (n = 830) and added

	HR (95% CI)	*P*-value
IHD (n = 651)		
Diabetes duration (increase of 1 year)	1.02 (1.004, 1.04)	0.021
Heart rate (increase of 1 beat/min)	1.02 (1.005, 1.03)	0.009
Ln(uACR)^a^	1.21 (1.08, 1.35)	0.001
Distal symmetrical polyneuropathy	1.29 (1.05, 1.57)	0.014
CAN category: none	1.00	
Possible	0.96 (0.66, 1.41)	0.851
Definite	1.00 (0.60, 1.68)	0.993
HF (n = 788)		
Age (increase of 1 year)	1.05 (1.02, 1.07)	< 0.001
Aboriginal descent	2.79 (1.51, 5.18)	0.001
Diabetes duration (increase of 1 year)	1.03 (1.01, 1.05)	0.011
Ln(uACR)^a^	1.33 (1.18, 1.49)	< 0.001
Distal symmetrical polyneuropathy	1.49 (1.19, 1.85)	< 0.001
Peripheral arterial disease	1.66 (1.11, 2.48)	0.013
Ischaemic heart disease	1.95 (1.32, 2.88)	0.001
CAN category: None	1.00	
Possible	0.94 (0.61, 1.46)	0.789
Definite	1.27 (0.78, 2.06)	0.342
All-cause mortality (n = 827)		
Age (increase of 1 year)	1.07 (1.05, 1.09)	< 0.001
Male sex	1.55 (1.12, 2.15)	0.008
Aboriginal descent	8.82 (3.20, 24.3)	< 0.001
Other European ethnic background	0.36 (0.16, 0.79)	0.011
Antidepressant therapy	3.06 (1.29, 7.26)	0.011
Angiotensin receptor blocker use	1.42 (1.03, 1.95)	0.031
eGFR < 30 mL/min/1.73m^2^	2.82 (1.40, 5.69)	0.004
Distal symmetrical polyneuropathy	1.14 (0.94, 1.38)	0.174
Cerebrovascular disease	1.67 (1.03, 2.73)	0.039
HF	2.42 (1.42, 4.13)	0.001
CAN category: None	1.00	
Possible	1.47 (1.01, 2.14)	0.046
Definite	2.42 (1.60, 3.67)	< 0.001
Time-varying covariates		
Aboriginal descent	0.42 (0.24, 0.75)	0.003
Antidepressant therapy	0.62 (0.39, 0.99)	0.047

Hazard ratios (HR) and 95% confidence intervals (95% CI) are shown. The proportional hazards assumption was violated in the model for all-cause mortality but not for IHD or HF

^a^A 2.72-fold increase in urinary albumin:creatinine ratio corresponds to an increased risk of 1 in ln(urinary albumin:creatinine)

### Incident heart failure

The characteristics of the 119 FDS2 participants (15.0%) who had a HF event during follow-up and those who did not are summarised in Table 5. The Kaplan–Meier curves of HF events during a mean ± SD follow-up period of 10.1 ± 2.8 (range 0.0–12.6) years (equivalent to 7992 person-years) are shown in Fig. 1 (middle panel). There was a significant difference between the three groups (log rank test *P* < 0.001), with definite CAN different from both the no CAN group (*P* < 0.001) and possible CAN group (*P* = 0.002) in unadjusted pairwise comparisons. In Cox proportional hazards modelling, increasing age, Aboriginal ethnic background, longer diabetes duration, a higher uACR, DSPN, PAD and a prior history of IHD were independent predictors of incident HF events but CAN category did not add significantly to the model (see Table 4).

**Table 5:** Baseline characteristics of FDS2 type 2 diabetes participants attending for their first ANS examination between May 2009 and November 2012 by incident hospitalisation for/with heart failure or death from heart failure to 31 December 2021, excluding those with prior hospitalisation for/with heart failure

	No hospitalisation for/with or death from HF	Incident hospitalisation for/with or death from HF^b^	*P*-value
N (%)	675 (85.0)	119 (15.0)	
CAN group (%)			0.002
None	53.8	43.7	
Possible	33.6	31.1	
Definite	12.6	25.2	
MCR (n = 794)	21.3 [12.1–33.3]	16.1 [7.9–25.3]	< 0.001
E:I ratio (n = 794)	1.13 [1.08–1.19]	1.11 [1.06–1.17]	0.008
Valsalva ratio (n = 775)	1.47 [1.31–1.68]	1.38 [1.22–1.52]	< 0.001
30:15 Stand ratio (n = 794)	1.15 [1.08–1.23]	1.12 [1.06–1.18]	0.005
Orthostatic hypotension (%)	12.5	8.4	0.223
Age (years)	61.4 ± 10.2	66.5 ± 11.2	< 0.001
Male (%)	56.4	55.5	0.842
Education beyond primary level (%)	93.2	93.1	> 0.999
Not fluent in English (%)	6.4	8.4	0.425
Ethnic background (%)			0.050
Anglo-Celt	53.3	50.4	
Southern European	11.1	9.2	
Other European	7.4	6.7	
Asian	4.9	3.4	
Aboriginal	5.9	15.1	
Mixed/other	17.3	15.1	
Smoking status (%)			0.306
Never	52.8	51.7	
Ex-	37.2	33.6	
Current	10.0	14.7	
Alcohol consumption (standard drinks^a^/day)	0.3 [0–1.5]	0.1 [0–0.8]	0.023
Antidepressant use (%)	14.7	15.1	0.889
Tricyclic antidepressants	3.3	4.2	0.583
Selective serotonin reuptake inhibitors	8.3	6.7	0.715
Age at diabetes diagnosis (years)	52.9 ± 11.0	52.5 ± 13.6	0.764
Diabetes duration (years)	6.1 [2.9–13.1]	14.0 [5.8–19.2]	< 0.001
Diabetes treatment (%)			0.001
Diet/exercise	24.4	16.2	
Oral agents ± non-insulin injectables	56.0	47.9	
Insulin alone	3.1	6.8	
Insulin ± oral agents ± non-insulin injectables	16.5	29.1	
HbA_1c_ (%)	6.9 [6.2–7.7]	7.1 [6.4–8.2]	0.032
HbA_1c_ (mmol/mol)	52 [44–61]	54 [46–66]	0.032
Fasting serum glucose (mmol/L)	7.4 [6.5–9.1]	7.5 [6.3–9.3]	0.805
ABSI (m^11/6^ kg^−2/3^)	0.081 ± 0.005	0.082 ± 0.005	< 0.001
BMI (kg/m^2^)	31.9 ± 6.1	31.6 ± 6.6	0.663
Pulse rate (beats/min)	70 ± 11	70 ± 13	0.573
Supine systolic blood pressure (mmHg)	141 ± 19	150 ± 23	< 0.001
Supine diastolic blood pressure (mmHg)	81 ± 11	80 ± 13	0.796
Antihypertensive medication (%)	68.1	77.3	0.052
Angiotensin converting enzyme inhibitors	31.9	38.7	0.169
Angiotensin receptor blockers	32.9	40.3	0.117
Beta-blockers	13.9	26.1	0.002
Calcium channel blockers	20.9	35.3	< 0.001
Diuretics	24.1	30.3	0.169
Total serum cholesterol (mmol/L)	4.3 ± 1.0	4.4 ± 1.7	0.569
Serum HDL-cholesterol (mmol/L)	1.19 ± 0.30	1.23 ± 0.35	0.143
Serum triglycerides (mmol/L)	1.5 (0.9–2.5)	1.6 (0.9–2.8)	0.349
Lipid-lowering medication (%)	66.7	74.8	0.089
uACR (mg/mmol)	2.3 (0.7–7.1)	5.9 (1.1–32.4)	< 0.001
eGFR (mL/min/1.73m^2^)	85 ± 18	79 ± 21	0.002
eGFR categories (%)			0.003
≥ 90 mL/min/1.73m^2^	45.6	31.9	
60–89 mL/min/1.73m^2^	45.2	48.7	
45–59 mL/min/1.73m^2^	5.6	12.6	
< 45 mL/min/1.73m^2^	3.6	6.7	
Distal symmetrical polyneuropathy (%)	30.3	45.8	0.001
Peripheral arterial disease (%)	16.8	35.3	< 0.001
Prior hospitalisation for IHD (%)	15.4	36.1	< 0.001
Prior hospitalisation for cerebrovascular disease (%)	3.9	10.9	0.004

^a^1 standard drink = 10 U ethanol; ^b^two participants with unknown cause of death excluded from this analysis

### All-cause mortality

The characteristics of the 162 FDS2 participants (19.5%) who died during follow-up and those who did not are summarised in Table 6. The Kaplan–Meier curves of HF events during a mean ± SD follow-up period of 10.5 ± 2.4 (range 0.2–12.6) years (equivalent to 8,684 person-years) are shown in Fig. 1 (lower panel). There was a significant difference between the three groups (log rank test *P* < 0.001), with both possible (*P* = 0.015) and definite (*P* < 0.001) CAN significantly different from the no CAN group, and possible CAN significantly different from definite CAN (*P* < 0.001), in unadjusted pairwise comparisons. In Cox proportional hazards modelling, increasing age, male sex, Aboriginal descent, Other European ethnic background, use of antidepressant therapy and of angiotensin receptor blockers, an eGFR < 30 mL/min/1.73 m^2^, DSPN, and a prior history of both cerebrovascular disease and HF were independent predictors of mortality. CAN category added significantly to the model at the expense of DSPN (see Table 4).

**Table 6:** Baseline characteristics of FDS2 participants with type 2 diabetes by all-cause mortality at end of follow-up

	Alive	Deceased	*P*-value
N	668 (80.5)	162 (19.5)	
CAN group (%)			< 0.001
None	54.6	35.8	
Possible	33.4	35.2	
Definite	12.0	29.0	
MCR (n = 830)	21.5 [12.9–33.9]	11.8 [6.8–23.7]	< 0.001
E:I ratio (n = 830)	1.13 [1.08–1.19]	1.11 [1.06–1.15]	< 0.001
Valsalva ratio (n = 809)	1.47 [1.31–1.68]	1.37 [1.22–1.51]	< 0.001
30:15 Stand ratio (n = 829)	1.15 [1.09–1.23]	1.12 [1.05–1.18]	< 0.001
Orthostatic hypotension (%)	12.6	8.7	0.220
Age (years)	60.9 ± 10.2	68.0 ± 9.7	< 0.001
Male (%)	54.3	63.0	0.052
Education beyond primary level (%)	93.3	90.1	0.178
Not fluent in English (%)	6.9	6.2	0.862
Ethnic background (%)			0.093
Anglo-Celt	51.2	59.9	
Southern European	11.4	8.0	
Other European	8.4	4.3	
Asian	5.1	1.9	
Aboriginal/TSI	7.6	8.6	
Mixed/other	16.3	17.3	
Smoking status (%)			0.359
Never	53.1	48.1	
Ex-	35.6	41.8	
Current	11.3	10.1	
Alcohol consumption (standard drinks^a^/day)	0.3 [0–1.2]	0.1 [0–1.5]	0.106
Antidepressant use (%)	13.5	19.8	0.048
TCAs	2.8	4.9	0.213
SSRIs	7.9	8.0	> 0.999
Age at diabetes diagnosis (years)	52.2 ± 11.0	55.8 ± 12.8	0.001
Diabetes duration (years)	6.5 [3.0–14.0]	11.0 [4.5–17.1]	< 0.001
Diabetes treatment (%)			0.223
Diet/exercise	23.6	21.3	
Oral agents ± non-insulin injectables	55.2	50.0	
Insulin alone	3.8	4.4	
Insulin ± oral agents ± non-insulin injectables	17.4	24.4	
HbA_1c_ (%)	6.9 [6.3–7.8]	6.8 [6.3–7.7]	0.347
HbA_1c_ (mmol/mol)	52 [45–62]	51 [45–61]	0.347
Fasting plasma glucose (mmol/L)	7.4 [6.4–9.2]	7.5 [6.2–9.0]	0.199
ABSI (m^11/6^ kg^−2/3^)	0.081 ± 0.005	0.082 ± 0.005	< 0.001
BMI (kg/m^2^)	32.1 ± 6.2	31.3 ± 6.5	0.138
Pulse rate (bpm)	70 ± 11	70 ± 12	0.391
Supine SBP (mmHg)	142 ± 20	145 ± 20	0.032
Supine DBP (mmHg)	81 ± 12	79 ± 12	0.016
On BP-lowering medication (%)	67.5	80.2	0.002
ACE-I	33.2	37.0	0.357
ARB	31.1	44.4	0.002
Beta-blockers	15.0	27.2	< 0.001
Calcium channel blockers	20.7	32.1	0.002
Diuretics	23.8	34.6	0.007
Total serum cholesterol (mmol/L)	4.3 ± 1.0	4.3 ± 1.5	0.840
HDL-cholesterol (mmol/L)	1.19 ± 0.30	1.19 ± 0.34	0.806
Serum triglycerides (mmol/L)	1.5 (0.9–2.6)	1.5 (0.9–2.6)	0.958
On lipid-lowering medication (%)	67.2	71.0	0.399
Urinary albumin:creatinine ratio (mg/mmol)	2.5 (0.7–9.3)	3.9 (1.0–15.2)	< 0.001
eGFR (mL/min/1.73m^2^)			
eGFR (CKD-EPI) stages (ml/min/1.73m^2^)			< 0.001
≥ 90	46.8	25.9	
60–89	45.2	47.5	
45–59	5.3	13.6	
30–44	2.1	7.4	
< 30	0.6	5.6	
Distal symmetrical polyneuropathy (%)	29.8	44.1	< 0.001
Peripheral arterial disease (%)	17.7	30.9	< 0.001
Prior hospitalisation for IHD (%)	16.8	35.8	< 0.001
Prior hospitalisation for cerebrovascular disease (%)	3.4	11.7	< 0.001
Prior hospitalisation for HF (%)	2.5	10.5	< 0.001

^a^1 standard drink = 10 U ethanol

### Relationship between individual CAN tests and outcome

Individual CART test results were included as continuous variables in separate Cox models of the three outcomes in place of CAN category. This allowed use of data from participants of all ages and those whose incomplete CART testing precluded CAN categorisation. Two models for each variable were constructed, the first involved participants in whom CAN category was determined (n = 830) and the second utilised available data from the 1254 participants who underwent CAN testing. None of the CART variables was a significant predictor of incident IHD or HF after adjusting for the most parsimonious model. The results of analyses for all-cause mortality are shown in Table 7. The proportional hazards assumption was violated in the model involving CAN-categorised participants, with the effects of Aboriginal descent and antidepressant therapy attenuating over time. For the second model, there were 1,101 participants with an MCR and E:I ratio (age range 17–95 years), 1100 with a 30:15 stand ratio (age range 17–95 years), and 904 with a Valsalva ratio (age range 17–89 years). The proportional hazards assumption was violated in this latter model, with the effect of age strengthening over time. In both models, MCR showed a significant inverse association with all-cause death.

**Table 7:** Most parsimonious Cox models of independent predictors of all-cause death in the FDS2 type 2 diabetes cohort with CAN category defined (n = 830) and valid CARTs variables as continuous variables added and retained if statistically significant (up to n = 1101)

	HR (95% CI)	*P*-value
CAN category defined and valid CART results		
All-cause mortality (n = 827)		
Age (increase of 1 year)	1.06 (1.04, 1.08)	< 0.001
Male sex	1.55 (1.12, 2.15)	0.008
Aboriginal descent	8.95 (3.26, 24.6)	< 0.001
Other European background	0.41 (0.19, 0.90)	0.026
Antidepressant therapy	3.40 (1.44, 8.00)	0.005
Angiotensin receptor blocker use	1.38 (1.001, 1.89)	0.049
eGFR < 30 mL/min/1.73m^2^	3.03 (1.51, 6.06)	0.002
Distal symmetrical polyneuropathy	1.15 (0.96, 1.39)	0.130
Cerebrovascular disease	1.70 (1.04, 2.77)	0.033
HF	2.31 (1.36, 3.93)	0.002
MCR (increase of 1.0)	0.98 (0.97, 0.99)	0.001
Time-varying covariates		
Aboriginal descent	0.43 (0.24, 0.76)	0.004
Antidepressant therapy	0.57 (0.38, 0.98)	0.043
Participants of all ages and valid CART results		
All-cause mortality (n = 1091)		
Age (increase of 1 year)	1.02 (0.998, 1.05)	0.070
Male sex	1.45 (1.13, 1.87)	0.004
Aboriginal descent	2.61 (1.58, 4.31)	< 0.001
Antidepressant therapy	1.47 (1.08, 2.01)	0.016
Ln(uACR)^a^	1.10 (1.004, 1.21)	0.042
eGFR < 30 mL/min/1.73m^2^	3.20 (1.88, 5.44)	< 0.001
Distal symmetrical polyneuropathy	1.31 (1.09, 1.57)	0.003
Peripheral arterial disease	1.42 (1.09, 1.85)	0.010
HF	1.67 (1.11, 2.50)	0.014
MCR (increase of 1.0)	0.99 (0.98, 0.998)	0.021
Time-varying covariates		
Age (increase of 1 year)	1.03 (1.02, 1.05)	< 0.001

Hazard ratios (HR) and 95% confidence intervals (95% CI) are shown

^a^A 2.72-fold increase in urinary albumin:creatinine ratio corresponds to an increased risk of 1 in ln (urinary albumin:creatinine)

## Discussion

The present study involving representative, community-based people with type 2 diabetes followed for an average of 10 years, showed that definite CAN was significantly associated with incident IHD and HF compared to both no CAN and possible CAN in survival analyses. However, when CAN category was included in multivariable models of these two incident events, it did not add to other independent predictors. Survival analysis also showed that there was a graded increase in risk of all-cause mortality from no CAN through possible CAN to definite CAN which was observed in multivariable analysis after adjustment for confounders. The only individual CART test predictive of all-cause death was MCR. Taken together, these findings question the need for screening for CAN, as has been suggested [2, 7], as part of routine care of type 2 diabetes, especially since the gold standard CART evaluation is demanding for both patients and staff [6]. In addition, the present data suggest that around one-third of patients will either be ineligible because of age or they will, for various reasons, be unable to complete a valid CART assessment.

In a recent narrative review, the prevalence of CAN in type 2 diabetes was reported as between 31 and 73% [3], but a subsequent Danish primary care study found a much lower prevalence of 9% after 6 years of screening-detected diabetes [4]. Our prevalence of definite CAN was intermediate between these values at 15.3%. Since almost all of the studies in the narrative review were conducted in secondary care [3], it is likely that our community-based cohort had less at-risk participants than secondary care studies with referred patients but more than in a pure primary care context [4]. The CAN risk factor profile in our FDS2 participants included independent variables that have been reported previously including BMI, longer diabetes duration, resting tachycardia (reflecting increased sympathetic tone [1]), as well as a prior history of HF (another potential manifestation of sympathetic overactivity and neurohormonal dysregulation [29]). A previously recognised positive association with antidepressant use in the general population [30] was confirmed in the present case of type 2 diabetes. These considerations suggest that, despite the exclusion of FDS2 participants who were recruited before CAN testing was available as part of baseline assessment, those (largely elderly) whose CART data could not be assessed against reference ranges and those in whom valid CART data could not be collected, our final sample of 830 generated representative data and was amongst the larger of studies reporting CAN prevalence [3, 4] and prognosis [7].

Our Kaplan–Meier analyses showed a significant relationship between CAN category and incident IHD. This was consistent with the results of a recent meta-analysis [7] in which there was significant heterogeneity, reflecting a variety of sample sizes, participant sources including people with type 1 diabetes and those with type 2 diabetes selected for clinical trials [8, 31, 32], and methods of diagnosing CAN which ranged from full CARTs to change in heart rate on standing [32] and heart rate variability (HRV) and QT index on resting electrocardiography [8]. Our multivariable analysis showed HRs for possible and definite CAN that were close to unity in the presence of other recognised predictors of incident IHD (longer diabetes duration, higher heart rate and increased uACR [2]). It is possible that relatively intensive CVD risk factor management in FDS2 paralleling trends in other high income countries [9, 10] (for example, approximately two-thirds of our participants were taking renin-angiotensin blocking drugs and statins) attenuated both the risk of CAN and its effect on CVD outcomes found in earlier studies, most of which were published before the first FDS2 patient was assessed for CAN [7]. In addition, we excluded participants with a history of IHD at baseline which may not have been the case in at least some of the studies with consequently higher risk samples in the meta-analysis, a consideration that may have contributed to the heterogeneity observed.

There are limited data assessing the relationship between CAN complicating type 2 diabetes and incident HF. In a report from the ACCORD trial involving trial participants with high CVD risk followed for a mean of 4.9 years, those with CAN defined from quartiles of HRV had a 2.7-fold greater risk of HF in adjusted analyses [33]. In our community-based participants assessed using CART, there was a significant relationship between definite CAN and incident HF in Kaplan–Meier analysis, with a more than doubling of the risk at 10 years. However, as with incident IHD, multivariable analysis showed HRs for possible and definite CAN that were close to unity in the presence of other recognised significant independent predictors of incident HF (older age, Aboriginal descent, longer diabetes duration, higher uACR, DSPN, and prior IHD) [34, 35]. We hypothesise that relatively intensive CVD risk factor management in FDS2, especially the large proportion of our participants who were taking renin-angiotensin blocking drugs, contributed to the lack of a significant association in a cohort studied before the widespread availability in Australia of the newer blood glucose-lowering agents (sodium-glucose co-transporter-2 inhibitors and glucagon-like peptide 1 receptor agonists) with beneficial effects on HF. As with IHD, we also excluded those with a history of HF at baseline.

In both Kaplan–Meier and Cox proportional hazard analyses, there was a significant and graded relationship between CAN category and all-cause mortality, with definite CAN associated with a more than doubling of risk at 10 years after adjustment for the presence of other recognised significant independent predictors of death (increasing age, male sex, Aboriginal descent, antidepressant therapy, eGFR < 30 mL/min/1.73m^2^, DSPN, and prior cerebrovascular disease and HF) [36–38]. In a recent meta-analysis, the unadjusted risk ratio for death was more than three-fold increased in people with CAN [7]. By far the largest contributor of participants and events in this study was the ACCORD trial [8] in which CAN was associated with a 1.55–2.14-fold risk of mortality after full adjustment for confounders, the risk ratio range reflecting three different methods of CAN ascertainment. However, the ACCORD participants were selected as having high CVD risk, the definition and staging of CAN was based on electrocardiographic indices with or without the presence of DSPN, and the follow-up duration was relatively short (3.5 years) [8]. Although these considerations complicate comparisons with the present study, the ACCORD findings are largely consistent with those of the present study.

The use of a representative, community-based sample in the present study may have masked sub-groups of people with type 2 diabetes in whom CAN has independent predictive value for IHD and HF. Such a sub-group may comprise those who are at high cardiovascular risk or who have established CVD [8, 33]. Nevertheless, in the Detection of Ischemia in Asymptomatic Diabetics (DIAD) study involving participants with type 2 diabetes without known heart disease [39], the incidence of the composite clinical outcome of cardiac death, acute coronary syndromes, HF, or coronary revascularization over 5 years was significantly increased in those in the lowest quartile of the Valsalva heart rate ratio (hazard ratio 1.60) amongst a range of tests of autonomic heart rate/blood pressure responses and power spectral analysis of HRV. The results of these studies should be interpreted against the heterogeneity in meta-analysis of prognostic studies of CAN [7] which highlights the influence of sample selection and CAN assessment methods. In addition, the duration of follow-up may be an important consideration since there is evidence that CART indices of autonomic dysfunction attenuate over time [4]. Our mean 10-year follow-up may have captured the effect of this on incident IHD and HF events compared to the ≤ 5 year follow-up in studies such as ACCORD and DIAD [8, 33, 39].

We found evidence that, of the individual CARTs performed, only reduced HRV as assessed from MCR was independently associated with all-cause mortality. MCR is one of the more robust CARTS as it is not influenced by changes in heart rate and presence of extrasystoles [40]. HRV has been shown to be a strong predictor of death in general population studies independently of cardiac or all-cause mortality and other clinical covariates [41]. It is a nonspecific predictor of mortality which reflects central-autonomic moment-to-moment adaption of somatic responses and emotional appraisal to maintain homeostasis and adapt to environmental stimuli [42]. In the context of diabetes, detection of a low HRV through measures such as MCR should prompt consideration of improved lifestyle factors including exercise [43].

The present study had limitations. As acknowledged, we excluded around one in 14 potentially eligible participants because of their age. Although we could have included them by using fixed thresholds for the various CART tests, as has been done in previous studies [20, 21, 44], there are important effects of age and sex on CART reference ranges [23, 45]. In any case, we included CART results as continuous variables and found that they were not predictive of endpoints apart from MCR for all-cause death. The presence of orthostatic hypotension in addition to abnormal heart rate test results identifies severe or advanced CAN [1]. However, probably due to the high percentage of our participants taking at least one antihypertensive medication (69.5%), a recognised confounding variable [1, 25], severe CAN was not an independent predictor in any of our multivariable analyses (data not shown). In addition, anaemia is a recognised complication of type 2 diabetes [46] and can contribute to orthostatic hypotension [47]. The major strengths of our study include its relatively large, community-based sample with rich phenotypic data, the use of gold standard CAN tests, and long follow-up for outcomes of interest.

## Conclusions

The present study has provided no evidence that either possible or definite CAN assessed by the range of recommended CARTs is an independent predictor of incident IHD or HF during relatively long-term follow-up in community-based people with type 2 diabetes. Possible and especially definite CAN were associated with all-cause mortality. There was evidence that this was mediated through a reduced MCR which has also been found to be a nonspecific adverse prognostic indicator in general population studies. Although the clinical value of routine assessment of CAN in type 2 diabetes is questionable as a result of our findings, the presence of CAN should still be established where typical symptoms (including light-headedness, weakness, palpitations and syncope on standing) or other features of autonomic neuropathy such as gastroparesis are present. The results could guide use of fludrocortisone and midodrine, and help tailor use of established therapies for CVD and glycaemic control [2]. In resource-limited settings, or where there are physical impairments to full CART testing, single diagnostic tests could be employed such as heart rate variation on deep breathing [6] or analysis of ten-second resting electrocardiographic tracings [8].

### Supplementary Information

**Additional file 1: Table S1.** Baseline characteristics of FDS2 type 2 diabetes participants attending their first ANS examination between May 2009 and November 2012 categorised by eligibility (valid CART data and aged 20 to 80 years old).

## Data Availability

Some outcome data supporting the findings of this study are available from the Western Australian Department of Health, but restrictions apply to the availability of these data, which were used under strict conditions of confidentiality for the current study, and so are not publicly available. Data are however available from the authors upon reasonable request and with permission of Western Australian Department of Health.

## Abbreviations

ABSI: A body shape index
ACCORD: Action to Control Cardiovascular Risk in Diabetes
BMI: Body mass index
CAN: Cardiac autonomic neuropathy
CVD: Cardiovascular disease
CART: Cardiovascular reflex test
CHD: Coronary heart disease
DIAD: Detection of Ischemia in Asymptomatic Diabetics
DSPN: Distal symmetrical polyneuropathy
eGFR: Estimated glomerular filtration rate
E:I: Expiration to inspiration ratio
FDS2: Fremantle Diabetes Study Phase II
HF: Heart failure
HMDC: Hospital Morbidity Data Collection
IHD: Ischaemic heart disease
MCR: Mean circular resultant
WA: Western Australia
WADLS: WA Data Linkage System
