# Supplementary material for: Prevalence and prognostic significance of cardiac autonomic neuropathy in community-based people with type 2 diabetes: the Fremantle Diabetes Study Phase II
Source: Cardiovasc Diabetol. 2024 Mar 18;23:102. doi: 10.1186/s12933-024-02185-3 (PMC10949593; doi:10.1186/s12933-024-02185-3)
Supplement: Supplementary file 1 — Additional file 1: Table S1. Baseline characteristics of FDS2 type 2 diabetes participants attending their first ANS examination between May 2009 and November 2012 categorised by eligibility (valid CART data and aged 20 to 80 years old). [file 12933_2024_2185_MOESM1_ESM.docx]

**Table S1.** Baseline characteristics of FDS2 type 2 diabetes participants attending their first ANS examination between May 2009 and November 2012 categorised by eligibility (valid CART data and aged 20 to 80 years old).

|  | **Not eligible** | **Eligible** | ***P*-value** |
| --- | --- | --- | --- |
| N | 424 (33.8) | 830 (66.2) |  |
| Orthostatic hypotension (%) | 11.4 | 11.9 | 0.853 |
| Age (years) | 71.0±10.6 | 62.3±10.5 | **<0.001** |
| Male (%) | 45.8 | 56.0 | **<0.001** |
| Education beyond primary level (%) | 82.7 | 92.6 | **<0.001** |
| Not fluent in English (%) | 13.7 | 6.7 | **<0.001** |
| Ethnic background (%) |  |  | 0.053 |
| Anglo-Celt | 55.2 | 52.9 |  |
| Southern European | 13.7 | 10.7 |  |
| Other European | 8.3 | 7.6 |  |
| Asian | 5.2 | 4.5 |  |
| Aboriginal/TSI | 4.0 | 7.8 |  |
| Mixed/other | 13.7 | 16.5 |  |
| Smoking status (%) |  |  | 0.142 |
| Never | 57.6 | 52.1 |  |
| Ex- | 33.9 | 36.8 |  |
| Current | 8.5 | 11.1 |  |
| Alcohol consumption (standard drinks/day) | 0.1 [0-0.8] | 0.3 [0-1.5] | **<0.001** |
| Antidepressant use (%) | 17.0 | 14.7 | 0.322 |
| TCAs | 5.9 | 3.3 | **0.035** |
| SSRIs | 9.0 | 8.0 | 0.589 |
| Age at diabetes diagnosis (years) | 59.0±11.4 | 52.9±11.5 | **<0.001** |
| Diabetes duration (years) | 11.2 [4.1-17.9] | 7.1 [3.0-15.0] | **<0.001** |
| Diabetes treatment (%) |  |  | 0.635 |
| Diet | 22.2 | 23.2 |  |
| OGLMs^1^ ± non-insulin injectables | 54.2 | 54.2 |  |
| Insulin alone | 5.4 | 3.9 |  |
| Insulin ± OGLMs ± non-insulin injectables | 18.2 | 18.8 |  |
| HbA_1c_ (%) | 7.0 [6.4-7.9] | 6.9 [6.3-7.8] | 0.354 |
| Fasting plasma glucose (mmol/L) | 7.5 [6.4-9.2] | 7.4 [6.4-9.2] | 0.805 |
| ABSI (m^11/6^ /kg^2/3^) | 0.082±0.005 | 0.081±0.005 | **<0.001** |
| BMI (kg/m^2^) | 30.8±5.8 | 31.9±6.2 | **0.001** |
| Heart rate (bpm) | 70±12 | 70±12 | 0.825 |
| Supine SBP (mmHg) | 147±22 | 142±20 | **<0.001** |
| Supine DBP (mmHg) | 79±13 | 81±12 | **0.022** |
| On BP-lowering medication (%) | 79.0 | 70.0 | **<0.001** |
| ACE-I | 38.0 | 34.0 | 0.170 |
| ARB | 33.5 | 33.7 | 0.950 |
| Beta-blockers | 22.4 | 17.3 | **0.033** |
| Calcium channel blockers | 30.4 | 22.9 | **0.005** |
| Diuretics | 35.4 | 25.9 | **<0.001** |
| Total serum cholesterol (mmol/L) | 4.3±1.1 | 4.31±1.1 | 0.786 |
| HDL-cholesterol (mmol/L) | 1.26±0.35 | 1.19±0.31 | **<0.001** |
| Serum triglycerides (mmol/L) | 1.5 (0.9-2.5) | 1.5 (0.9-2.6) | 0.192 |
| On lipid-lowering medication (%) | 730 | 68.0 | 0.069 |
| Urinary albumin:creatinine ratio (mg/mmol) | 4.2 (1.0-17.7) | 2.7 (0.7-10.3) | **<0.001** |
| eGFR (CKD-EPI) stages (ml/min/1.73m^2^) |  |  | **<0.001** |
| ≥90 | 23.4 | 42.8 |  |
| 60-89 | 54.8 | 45.7 |  |
| 45-59 | 12.1 | 6.9 |  |
| 30-44 | 5.2 | 3.1 |  |
| <30 | 4.5 | 1.6 |  |
| Distal symmetrical polyneuropathy (%) | 45.5 | 32.6 | **<0.001** |
| Peripheral arterial disease (%) | 27.8 | 20.3 | **0.003** |
| Prior hospitalisation for ischaemic heart disease (%) | 28.1 | 20.5 | **0.003** |
| Prior hospitalisation for cerebrovascular disease (%) | 5.7 | 5.1 | 0.689 |
| Prior hospitalisation for heart failure (%) | 7.5 | 4.1 | **0.011** |
| CART components available (%): |  |  |  |
| E:I ratio (n=1040) | 210 (49.5) | 830 (100) | **<0.001** |
| MCR (n=1063) | 233 (55.0) | 830 (100) | **<0.001** |
| 30:15 Stand ratio n=(1061) | 232 (54.7) | 829 (99.9) | **<0.001** |
| Valsalva ratio (n=880) | 71 (16.7) | 809 (97.5) | **<0.001** |

^1^OGLMs oral glucose lowering medications
